# Surgical maculopathies: epiretinal membranes and full-thickness macular holes

**Published:** 2025-01-31

**Authors:** Anthony Hall

**Affiliations:** 1 Consultant Ophthalmologist: Hunter Eye Surgeons, Newcastle, Australia.

**Epiretinal membrane and full-thickness macular hole are common and treatable conditions of the retina. Left untreated, they can lead to profound loss of central vision, but with prompt and effective surgery, most patients will achieve significant improvements in visual acuity and distortion.**

Surgery offers hope to patients affected by epiretinal membrane and full-thickness macular hole, both of which result in loss of central vision. Epiretinal membranes and full-thickness macular holes are both associated with vitreous detachment, usually in people during their 60s or 70s. It is important to recognise them as they can affect vision, and effective treatment is available. They often coexist with cataract, which may make them difficult to detect and lead to disappointing visual outcomes from cataract surgery.

## How patients present

Patients with **epiretinal membranes** present with symptoms such as reduced central visual acuity, blurring, metamorphopsia (distortion of straight lines), aniseikonia (different-sized images) and loss of stereopsis. Vision loss is gradual and may not be noticed by patients.

Unlike epiretinal membranes, which come on quite slowly, patients with **full-thickness macular holes** present with fairly acute loss of central vision, as full-thickness macular holes occur quite rapidly. If a patient presents with reduced central vision and the lens is still relatively clear, it is mandatory to examine the macula and look for a full-thickness macular hole, as prompt diagnosis and referral can restore vision to normal.

Full-thickness macular hole is three times more common in women than in men. Epiretinal membrane is also slightly more common in women. Large population-based studies found a prevalence of epiretinal membrane of between 7% and 11.8% using fundus photographs, but this increased to 34% when optical coherence tomography (OCT) was used. Full-thickness macular hole is less common, occurring in between 0.2 to 3.3 per 1,000 population.^1^ As both these conditions are uncommon in patients under 60, their prevalence will be lower in countries with a younger population.

**How epiretinal membranes and macular holes develop**

As people get older, the vitreous gel in the eye starts to degenerate. The posterior surface of the gel separates from the back of the eye and moves forward, towards the front of the eye. This is known as a posterior vitreous detachment. As the gel moves forwards, it can tear the peripheral retina, which can lead to **retinal detachment**.

Sometimes, a posterior vitreous detachment can result in conditions that affect **central** vision: epiretinal membrane and full-thickness macular hole.

In **epiretinal membrane**, the cells left behind or deposited on the surface of the retina after the vitreous detaches proliferate and – as the tissue contracts – this causes distortion and thickening of the macula.

The detaching vitreous may also pull on the fovea. A combination of anterior-posterior traction and tangential traction from the elastic internal limiting membrane results in a separation of all the layers of the retina, right through to the photoreceptor layer (outer retina), known as a **full-thickness macular hole**. There is no loss of retinal tissue, but the separation of retinal tissue means a loss of central vision. This can be restored if the hole is closed.

## Epiretinal membrane

Epiretinal membrane may be primary or secondary. Primary epiretinal membrane occurs when there is no risk factor other than a posterior vitreous detachment. Secondary epiretinal membrane occurs in conjunction with other eye diseases, most commonly diabetic retinopathy, retinal vein occlusion, or retinal tears/detachment.^2^

Clinical grading, based on photographs or slit lamp examination, may be divided into cellophane maculopathy, which is an early translucent epiretinal membrane without distortion of the retina, and macular pucker, or preretinal fibrosis, which is a more advanced form of epiretinal membrane, causing distortion of the inner retina.

### History and examination

Enquire about diabetes, previous ocular trauma, and a history of flashing lights and floaters. Many patients with early epiretinal membrane are asymptomatic and the membrane may be identified at routine examination. Vision may be affected by traction, retinal oedema, or an opaque membrane.

**Figure 1:**
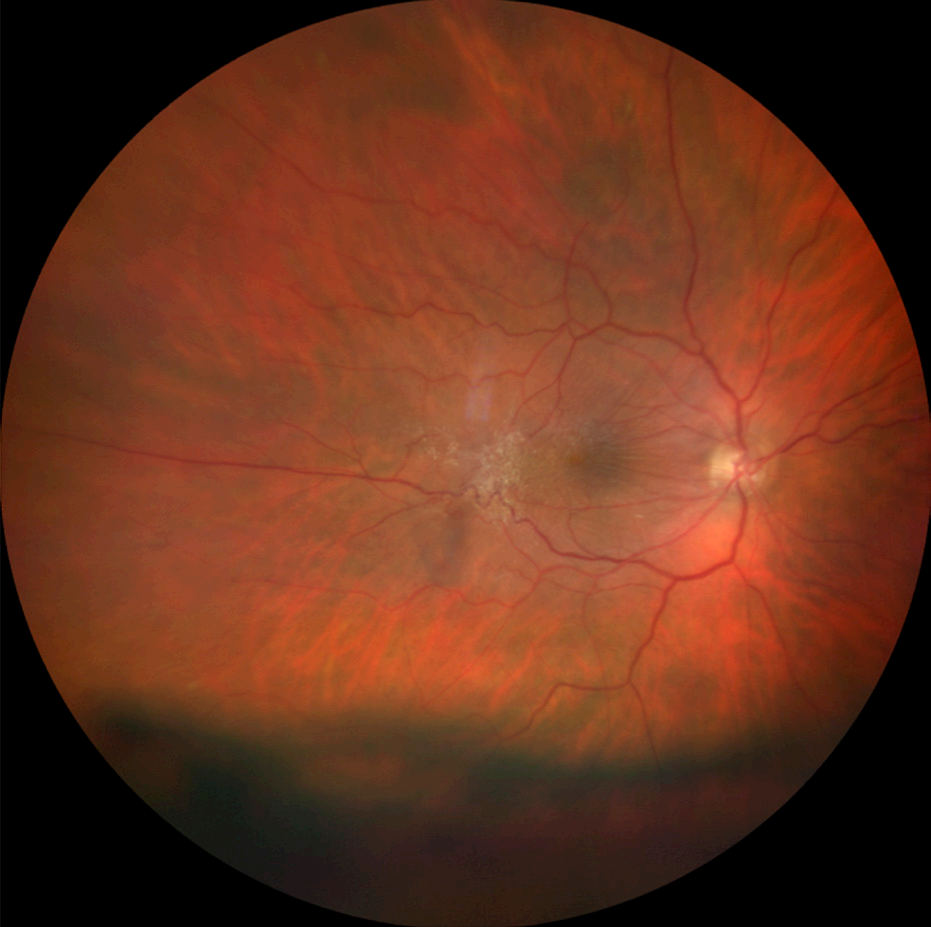
The epiretinal membrane is the white patch temporal to the fovea. This is an area of fibrous tissue which is contracting. The contraction has drawn the blood vessels of the superior and inferior arcades towards the membrane. Traction lines can be seen as folds in the retina, starting near the disc and crossing the fovea, going towards the epiretinal membrane.

**Figure 2:**
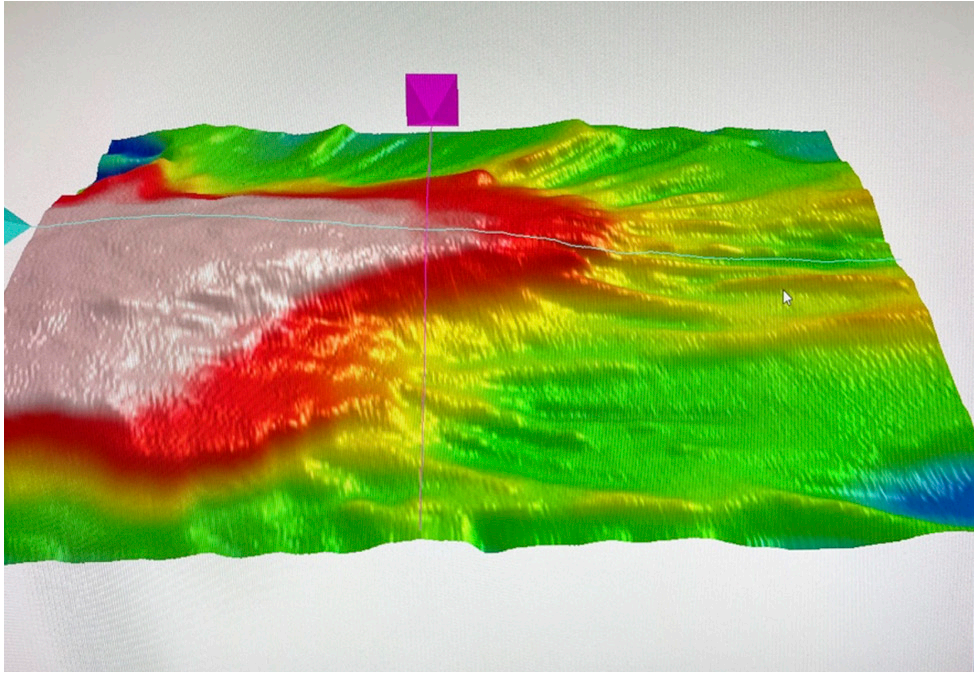
An OCT retinal thickness map demonstrates how the thick contracting membrane has elevated the retina temporally and is causing traction folds in the surrounding retina.

Early membranes can be seen as a glistening light reflex over the macula (Figure 1). As the membrane progresses, the retinal surface is wrinkled (Figure 2), and blood vessels become more tortuous or straightened. It may also be possible to detect cystoid macular oedema, loss of the foveal reflex, pseudoholes, and full-thickness macular holes.

© ANTHONY HALL CC BY-NC-SA 4.0

© ANTHONY HALL CC BY-NC-SA 4.0

### Investigations

Optical coherence tomography (OCT) examination is invaluable in diagnosing, managing, and following patients with epiretinal membrane and macular holes. It is able to detect associated conditions like lamellar macular holes (a foveal cavity with undermined edges and loss of foveal tissue) and foveoschisis (separation of the foveal layers).

### Treatment

Most patients with epiretinal membrane will not require any treatment. Progression of epiretinal membrane is uncommon, and, if the patient is asymptomatic, they may be discharged and advised to return if their vision gets worse. Surgery for epiretinal membrane is performed when patients’ visual loss or symptoms affect their activities of daily living. The goal of surgery is to stabilise or improve vision and to reduce metamorphopsia. If you are unsure if a patient needs to be referred, ask them “Do you close your bad eye in order to see better with the good eye?” If they do, it is worth referring them to a retinal surgery centre.

Surgery consists of pars plana vitrectomy and removal of the epiretinal membrane, which may be combined with peeling of the internal limiting membrane. The surgeon uses a blue dye to stain the membranes and then peels them away from the macula with microforceps.

### Complications

All patients having vitrectomy for full-thickness macular hole or epiretinal membrane should be told that they will develop a cataract. Indeed, many surgeons combine cataract extraction with the vitrectomy. Patients should be warned of the risk of retinal detachment, haemorrhage, endophthalmitis, and hypotony, but these complications are rare.

### Prognosis

On average, vision improves by two lines of visual acuity. The greatest improvement is seen in patients with poor pre-operative visual acuity, but the final visual acuity is better in those with a better pre-operative visual acuity. Patients also get relief from metamorphopsia. The reduced distortion improves binocular vision, making it easier for the two eyes to work together. Patients usually report an improvement in their vision-related quality of life, even if there is only a small improvement in visual acuity. Following surgery, visual improvement is slow and may take up to 3 years.

**Distinguishing pseudoholes from full-thickness holes**

A pseudohole is a circular defect in the epiretinal membrane overlying the fovea, and it resembles full-thickness macular hole.

The visual acuity is usually 6/36 or worse in full-thickness macular hole, but rarely worse than 6/24 in pseudoholes. Always check the peripheral retina carefully to detect untreated retinal tears, and look for signs of vein occlusion or diabetic eye disease.

## Full-thickness macular hole

**Figure 3:**
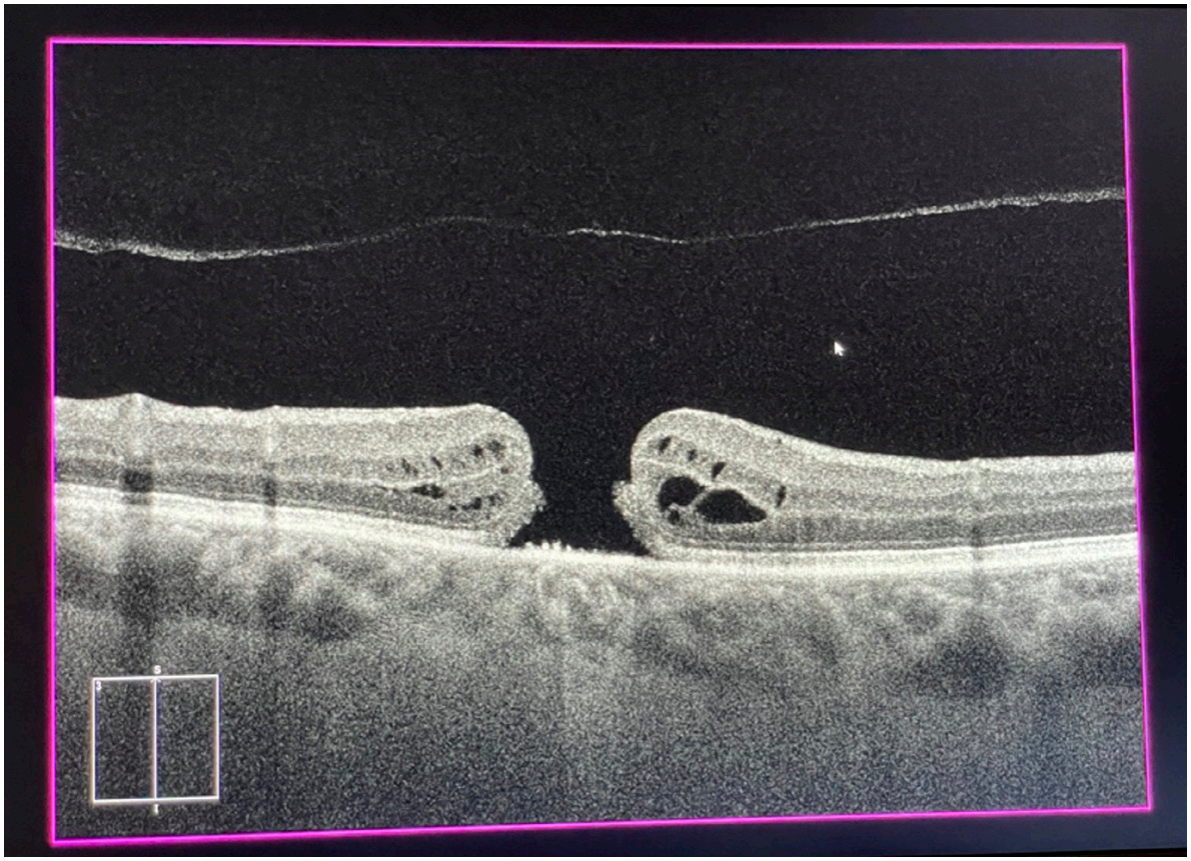
The HD OCT scan of the macular hole shows the separation of all the retinal layers at the fovea down to the RPE. The retina surrounding the hole has become oedematous with cystic spaces visible in the retinal layers.

Full-thickness macular hole is a defect in the fovea that involves all the neurosensory layers of the retina, from the internal limiting membrane down to the photoreceptor layer (Figure 3). Untreated, this is associated with severe central visual impairment. Full-thickness macular hole may be primary (caused during vitreoretinal detachment) or secondary. Causes of secondary full-thickness macular hole include trauma, severe myopia, and retinal detachment.

© ANTHONY HALL CC BY-NC-SA 4.0

### Diagnosis

**Figure 4:**
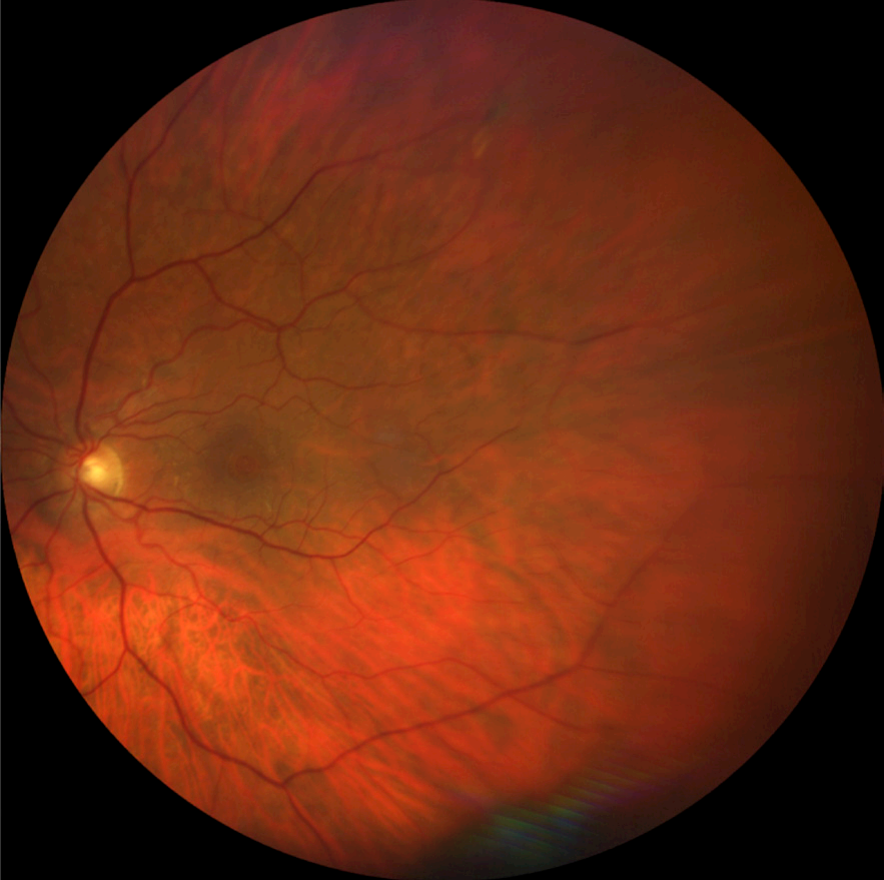
A colour photograph of the macular hole, showing how the central changes can be quite subtle. The cuff of oedema around the hole can be seen.

On presentation, depending on the duration of the macular hole, central visual acuity will range from 6/9 in very early, small holes, to counting fingers for long-standing, neglected holes. Macular holes can be diagnosed by careful slit lamp examination, but the signs are subtle (Figure 4). OCT scans are recommended.

© ANTHONY HALL CC BY-NC-SA 4.0

### Pathogenesis

As the vitreous starts to detach, it pulls on the fovea. A combination of this antero-posterior traction and tangential traction from the elastic internal limiting membrane leads to a full thickness hole. The edges of the hole become hydrated, leading to macular oedema.^3^

### Classification

The International Vitreomacular Traction Study Group proposed OCT-based staging (Duker et al., 2013). The two stages depend on the presence (early stage) of vitreomacular traction or its absence (late stage). Further subgroups, based on size measured at the narrowest point of the hole, are: small (< 250 µm), medium (> 250 µm and < 400 µm), large (>400 µm). However, recent studies show that there is little difference in prognosis between 350 µm and 450 µm holes. We should therefore probably regard holes from 250-500 µm as medium, and large holes as any hole >500 µm.

### Treatment

Patients with recent-onset full-thickness macular hole, in particular, will benefit from prompt diagnosis and referral.

Surgery for full-thickness macular hole is similar to that for epiretinal membrane, with a few important differences. A posterior vitreous detachment often has to be induced. Triamcinolone acetonide microparticles can stain the vitreous, making it easier to see. Inducing a posterior vitreous detachment carries a risk of retinal tears. The peripheral retina must be inspected very carefully and any tears treated.

Peeling the internal limiting membrane has been proven to increase the success rate of full-thickness macular hole surgery. In large and refractory holes (holes that have not closed with surgery), internal limiting membrane flaps can be used to plug the hole to improve healing. Once the vitrectomy is completed, the eye is filled with a gas. Short-acting gases such as SF6 are used with smaller holes, while longer-acting gases such as C3F8 improve the outcome with larger holes. Maintaining a strict face-down position after surgery is difficult for most patients, and we have found that – as long as the eyes are looking down all the time, the outcome for small to medium holes is excellent without the patient needing to maintain a face-down posture. There is little benefit from maintaining an eyes-down or face-down posture for longer than 3 days, even for patients with larger holes.

### Prognosis

The prognosis is excellent for small/medium holes with a short duration. An estimated 95% of holes smaller than 500 µm are closed with a single operation. The average vision improvement is approximately 0.5 LogMAR units, equivalent to a change from 6/36 to 6/12. Large, chronic holes are more difficult to treat, and even when they are successfully closed, the vision may not improve as much.

**From the field: Full-thickness macular hole surgery in Tanzania**

**Figure:**
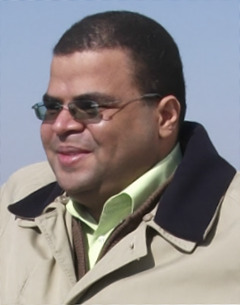

**William Makupa** is an ophthalmologist working in Moshi, Tanzania

Full-thickness macular hole is one the principal surgical retina pathologies and is the fifth most common indication for vitreoretinal surgery at Kilimanjaro Christian Medical Centre (KCMC) in Moshi, Tanzania.

At KCMC, we aim to offer full-thickness macular hole surgery to patients within a week of presentation. Optical coherence tomography has enhanced our clinical decision making, by helping us select the patients with the best prognosis.

In 2023, we performed 124 full-thickness macular hole operations out of a total of 746 vitreoretinal procedures. We prioritise patients in whom the hole has a diameter of 400 microns or less, as they have the best prognosis.

The surgical approach is pars plana vitrectomy, including induction of posterior vitreous detachment, staining with Brilliant Blue G 0.05% dye and peeling of the internal limiting membrane. Perfluoropropane 12% or sulphur hexaflouride 25% gas tamponade is used for all patients. The patients are asked to lie face down after the operation. Cataract surgery is scheduled at some time in the future. If there is an adequate view, it is common to see anatomical closure by day three postoperatively.

We found that 68% of full-thickness macular holes closed after one operation. A total of 63% patients achieved visual acuity of 6/60 or better at three months, compared to 39% preoperatively.

We also use a single pressurised canister of tamponade gas for multiple patients, thanks to aseptic protocols and gas regulators, which allow us to reuse the canisters. 

Over the last fourteen years, full-thickness macular hole surgery has become an essential part of the vitreoretinal service at KCMC. It has brought real benefits to our patients. Although we are operating in a resource-limited environment, these procedures can be done safely, effectively, and at an affordable cost to our community. 

However, many patients with full-thickness macular holes present late, after having symptoms for more than one year. Occasionally, the staining agent Brilliant Blue G 0.05% has also been out of stock.

## References

[1] Duker JS., Kaiser PK., Binder S. (2013). The International Vitreomacular Traction Study Group classification of vitreomacular adhesion, traction, and macular hole.. Ophthalmology..

[2] Fung AT., Galvin J., Tran T. (2021). Epiretinal membrane: A review.. Clin Exp Ophthalmol..

[3] Premi E., Donati S., Azzi L. (2022). Macular Holes: Main Clinical Presentations, Diagnosis, and Therapies.. J Ophthalmol..

